# Photochemistry of nitric oxide and *S*-nitrosothiols in human skin

**DOI:** 10.1007/s00418-020-01858-w

**Published:** 2020-03-11

**Authors:** Milena T. Pelegrino, André Paganotti, Amedea B. Seabra, Richard B. Weller

**Affiliations:** 1 Center for Natural and Human Sciences, Universidade Federal Do ABC, Av. dos Estados 5001, Santo André, SP CEP 09210-580 Brazil; 2 Laboratory of Materials and Mechanical Manufacture, Universidade Federal de São Paulo, Diadema, SP Brazil; 3 Centre for Inflammation Research, University of Edinburgh, 47 Little France Crescent, Edinburgh, EH16 4TJ UK

**Keywords:** Nitric oxide, *S*-nitrosothiols, Human skin, Ultraviolet irradiation, Photobiology

## Abstract

Nitric oxide (NO) is related to a wide range of physiological processes such as vasodilation, macrophages cytotoxicity and wound healing. The human skin contains NO precursors (NO_*x*_). Those are mainly composed of nitrite (NO_2_^−^), nitrate (NO_3_^−^), and *S*-nitrosothiols (RSNOs) which forms a large NO store. These NO_*x*_ stores in human skin can mobilize NO to blood stream upon ultraviolet (UV) light exposure. The main purpose of this study was to evaluate the most effective UV light wavelength to generate NO and compare it to each NO precursor in aqueous solution. In addition, the UV light might change the RSNO content on human skin. First, we irradiated pure aqueous solutions of NO_2_^−^ and NO_3_^−^ and mixtures of NO_2_^−^ and glutathione and NO_3_^−^ and *S*-nitrosoglutathione (GSNO) to identify the NO release profile from those species alone. In sequence, we evaluated the NO generation profile on human skin slices. Human skin was acquired from redundant plastic surgical samples and the NO and RSNO measurements were performed using a selective NO electrochemical sensor. The data showed that UV light could trigger the NO generation in skin with a peak at 280–285 nm (UVB range). We also observed a significant RSNO formation in irradiated human skin, with a peak at 320 nm (UV region) and at 700 nm (visible region). Pre-treatment of the human skin slice using NO_2_^−^ and thiol (RSHs) scavengers confirmed the important role of these molecules in RSNO formation. These findings have important implications for clinical trials with potential for new therapies.

**Electronic supplementary material:**

The online version of this article (10.1007/s00418-020-01858-w) contains supplementary material, which is available to authorized users.

## Introduction

Nitric oxide (NO) is a small but important molecule in mammalian biology. It is a free radical involved in a wide range of physiological processes, such as the control of blood pressure, neuronal communication, wound healing, and macrophage toxicity against pathogens, among others (Hirai et al. 2018; Nagasaka et al. 2018). NO was first described as the endothelium-derived relaxation factor (EDRF) because of its important role in the promotion of vasodilation (Ignarro et al. 1987). After this important discovery, NO has been linked with several other physiologic processes with promising implications in the biomedical field.

NO can be formed in a biological environment through enzymatic and nonenzymatic pathways. The enzymatic pathways involve the action of nitric oxide synthase enzymes (NOS), which have three isoforms, neuronal (nNOS), endothelial (nNOS) and inducible (iNOS) (Martínez-Ruiz et al. 2011; Seabra and Durán 2012; Seabra et al. 2015a, b). The nonenzymatic pathway of NO release is important in skin physiology and ultraviolet (UV) light exposure (Martínez-Ruiz et al. 2011; Weller 2016; Eilertsen et al. 2018).

NO has a short half-life in biological media and is oxidized with a half-life measured in seconds to more stable species collectively known as NO_*x*_. Human skin contains large stores of these NO_*x*_ (Paunel et al. 2005; Mowbray et al. 2009; Liu et al. 2014) which suggests an important physiological function (Liu et al. 2014; Weller 2016). The major components of this NO storage in the skin are nitrite ${(\mathrm{N}\mathrm{O}}_{2}^{-}$,) nitrate (${\mathrm{N}\mathrm{O}}_{3}^{-}$), and *S*-nitrosothiols (RSNOs) (Khan et al. 2003; Wright and Weller 2015; Weller 2016; Pelegrino et al. 2017a,b). RSNOs are defined in this study as a large group including *S*-nitrosoproteins, *S*-nitroso amino acids, *S*-nitroso-peptides and *S*-nitroso-sugars. An example of RSNO commonly found in human body is *S*-nitrosoglutathione (GSNO), which is formed by the nitrosation of the glutathione (GSH) (Gamcsik et al. 2012; Pelegrino et al. 2017b).

RSNO can be spontaneously decomposed and release NO (Williams 1999). The RSNO decomposition is influenced by its concentration, temperature, pH, light irradiation and presence of copper ions. (Williams 1999; Noble and Williams 2000; Zhelyaskov et al. 1998; de Souza et al. 2019). Light can be used to promote the photo-release of NO from RSNOs (Sexton et al. 1994; Zhelyaskov et al 1998; de Souza et al. 2019; Pelegrino et al. 2020). Pelegrino et al. 2020 irradiated a solution of GSNO at 1.0 mmol L^−1^ and showed a peak of NO production at 310 nm. Lopes-Oliveira et al. 2019 evaluated S-nitroso-mercaptosuccinic acid (S-nitroso-MSA, at initial concentration of 2 mmol L^−1^) decomposition with NO release irradiated with white light. They showed a burst of NO release in the first 15 min with approximately 80% of NO released and the establishment of a steady state at 1.76 ± 0.03 mmol L^−1^ of NO release (Lopes-Oliveira et al. 2019). In addition, copper ions and other metals can significantly increase the rates of RSNO decomposition (Noble and Williams 2000; Tuttle et al. 2019; Lutze et al. 1999). There are several studies attempting to create a more stable class of RSNOs (Khan et al. 2003; Eilertsen et al. 2018) or to incorporate RSNOs in materials/biomaterials to increase their stability (Kim et al. 2015; Pelegrino et al. 2017a, 2018; Ferraz et al. 2018).”

The NO_x_ stores in human skin can be mobilized to the blood stream upon sunlight irradiation, in particular, UV light (Hosenpud et al. 1985; Mowbray et al. 2009; Liu et al. 2014). The sunlight energy that reaches the earth’s outer atmosphere has UV (200–400 nm), visible (40–700 nm), and infrared (700–1000 nm) components. The UV light is divided as UVA (321–400 nm), UVB (280–320 nm) and UVC (200–280 nm) (Holick 2016; Marzulli and Maibach 2008). Liu et al. observed an increase in serum levels of NO_2_^−^ and RSNO after UVA irradiation on healthy volunteers (Liu et al. 2014). This mobilization of NO_*x*_ from skin to the blood stream led to arterial vasodilation and, as consequence, the decrease of the blood pressure by at least 5 mmHg for 30 min after the irradiation (Liu et al. 2014).

Cardiovascular diseases represent the leading cause of death globally (Santulli 2013) and high blood pressure underlies 16.5% of all deaths (Santulli 2013). There is a strong correlation between blood pressure and latitude, with people living close to the equator having lower blood pressure than those at higher latitudes (Weller 2016). UV-induced mobilization of NO_*x*_ from the skin to the systemic vasculature is a mechanism by which sunlight may exert beneficial effects on health (Weller 2016).

Although sunlight is a risk factor for skin cancer and aging, it has also been related to overall health benefits acting on the cardiovascular and immune systems (Wright and Weller 2015). Public health advice has traditionally focussed heavily on the dangers of sunlight exposure, but the most recent guidelines also call attention to possible health benefits (Geller et al. 2018).

The purpose of this study is to evaluate the most effective UV light wavelength to generate NO and compare it to each NO precursor in aqueous solution. In addition, the UV light might change the RSNO content on human skin.

## Materials and methods

### Chemicals

Glutathione (GSH), sodium nitrate (NaNO_3_), sodium nitrite (NaNO_2_), *N*-ethylmaleimide (NEM), sulfanilamide (SNM), and acetic acid were purchased from Sigma-Aldrich (St. Louis, MO, USA) and used without further purification. Aqueous solution preparations were carried out using analytical-grade water from a Millipore Milli-Q Gradient filtration system (resistivity below 18.2 MW cm^−1^ at 25.0 ºC).

### Synthesis of GSNO

GSNO was synthesized by the nitrosation of GSH. A stock GSH aqueous solution was prepared. To this end, the amount of 307.3 mg of GSH was dissolved in 10 mL of acetic acid 1% to obtain the final concentration of 100 mmol·L^−1^. Thus, we added 69 mg of NaNO_2_ into the GSH aqueous solution; the NaNO_2_ final concentration is equimolar of GSH (100 mmol·L^−1^). The final mixture was homogenized and incubated at low temperature (0–5 ºC) for 20 min. NaNO_2_ in slight acid media is dissociated and protonated forming nitrous acid (HNO_2_), which is responsible to nitrosate GSH forming GSNO. The formation of GSNO was confirmed through the detection of the characteristic absorbance peak of GSNO at 336 nm (ɛ = 980.0 mol^−1^·L cm^−1^), acquired using a Plate reader (BioTek, Synergy HT, Vermont, USA) (Silveira et al. 2016). No further purification method was necessary.

### Human skin

Human skin was acquired from Murrayfield private hospital at Edinburgh, Scotland, UK, from abdominoplasty surgery (South East Scotland Research Ethics Committee 1, reference 16-SS-0103). After the surgery, the skin was cleaned and transferred to the Queen Medical Research Institute (QMRI) facility, Edinburgh, Scotland, UK, on the same day. The fat layer of the skin was removed using a scalpel and scissors and the skin was cut with spherical shape using dermatological punch with 4 mm of diameter, leading to several skin slice samples with the same dimensions obtained from the same abdominal region. The skin slices were stored at – 20 ºC.

### Light source: monochromator

A monochromator (model 77200, ThermoOriel, Newport, UK) with a 1000 W deuterium and quartz lamps was used in this work, as described in the next sections. The output was directed via a 5 mm diameter liquid guide. Wavelengths and bandwidth (half-maximal bandwidth ~ 2.6 nm) calibrations were conducted using a Bentham double-grating spectroradiometer (3AP-CAL, OphirOptronics Ltd, Jerusalem, Israel).

### Real-time free NO release analyses

First, aqueous solutions of (i) NO_2_^−^ (0.05 mmol·L^−1^, pH 4.5), (ii) NO_3_^−^ (2.0 mmol·L^−1^, pH 4.5), (iii) a mixture of NO_2_^−^ (0.05 mmol·L^−1^, pH 4.5) + GSNO (1.0 mmol·L^−1^, pH 4.5), and (iv) a mixture of NO_3_^−^ (2.0 mmol·L^−1^, pH 4.5) + GSH (1.0 mmol·L^−1^, pH 4.5) were prepared. Real-time free NO generated from the solutions i–iv was measured using the nitric oxide meter (ISO-NO, NOMK2, World Precision Instruments, WPI, FL, USA) with an electrochemical sensor (ISONOP-4 mm), and compared with the free NO release from human skin (4 mm diameter skin slice immersed in 7 mL of water, *n* = 3). To this end, 7 mL of each group was transferred to a quartz cuvette with optical path of 20 mm. The electrochemical sensor was immersed into aqueous solutions i–iv or skin slice immersed in water and the signal of free NO production was electrochemically monitored, in the dark and under irradiation with the monochromator light source, described in item 2.4. The electrochemical sensor detects only free NO, since the sensor has a negative charged coating that allows NO pass through the coating while blocking other molecules such as NO_2_^−^ and NO_3_^−^ (Iverson et al. 2018). The NO sensor was inserted into the solutions (around 1 cm below the top of the quartz cuvette) and the light guide connected to the monochromator was positioned 90° relative to the NO sensor. The samples (solutions i–iv and skin slice immersed in water) were irradiated using liquid guide with narrow wavelengths centred at 250, 260, 270, 280, 290, 300, 310 and 320 nm (precision of 0.1 nm). Each wavelength was tested using *n* = 3 of skin slices. The samples were irradiated with different energies from 0 to 10 J cm^−2^ (Gibbsl et al. 1993). The collected data were adjusted with a determination coefficient (*R*^2^) of no less than 0.96. The mathematical procedure used is described with more details in the Supplementary Information section.

### Skin pre-treatments

To block the precursors of RSNO in human skin, skin slices (4 mm in diameter) were immersed into a solution of sulphanilamide (SNM) (0.5 mmol·L^−1^), an NO_2_^−^ scavenger or with *N*-ethylmaleimide (NEM) (0.5 mmol·L^−1^), a thiol scavenger, for 1 h at 25 ºC in the dark, as a pre-treatment. After the incubation period, the skin slices were transferred to a 12-well plate and irradiated at 320 and 700 nm at 3 J cm^−2^.

In an opposite way, to increase the levels of RSNO precursors in human skin, skin slices (4 mm of diameter) were immersed into a solution of NO_3_^−^ at different concentrations (50, 500, 1000 or 5000 µmol L^−1^) for 1 h at 25 ºC in the dark, as a pre-treatment. After the incubation period, the skin slices were transferred to a 12-well plate and irradiated at 320 and 700 nm at 3 J cm^−2^.

### RSNOs quantification in skin after irradiation

RSNO quantification was measured in skin slices under two distinct conditions: (i) non-pre-treated skin slices (4 mm), and (ii) pre-treated skin slices (4 mm), as described in Sect. 2.6. In both distinct conditions (i and ii), RSNO quantification was performed in the dark and after irradiation. The irradiation was performed by positioning the liquid guide on skin slices (4 mm) using narrow wavelengths centred at 290, 305, 300, 310, 320, 340, 400, 450, 500, 550, 600, 650, 700, 750 and 800 nm (precision of 0.1 nm) with different energies from 0 to 10 J cm^−2^.

After the irradiation, the skin slices were kept in an ice bath and the levels of RSNOs were quantified using the NO electrochemical sensor attached to the NO meter (described in Sect. 2.5) with the copper chloride method, which allows the quantification of NO release from RSNO reduction (Williams 1999; Noble and Williams 2000; Oliveira et al. 2016; Santos et al. 2016; Silveira et al. 2016). To this end, skin slices were homogenized using a glass tissue homogenizer of 15 cm^3^ adding 1.0 mL of Milli-Q water. After the homogenization, the samples were transferred to an Eppendorf flask and kept in an ice bath for the electrochemical quantification of RSNOs. The NO sensor was immersed in a solution of 10 mL of copper chloride II (CuCl_2_) at 0.1 mol L^−1^. A volume of 200 µL of the supernatant of the skin homogenate was added to the CuCl_2_ solution. The experiments were performed in duplicates of two independent experiments (*n* = 4) and the calibration curves were obtained with aqueous solutions of freshly prepared *S*-nitrosoglutathione (GSNO) (data not shown) (Oliveira et al. 2016).

### Statistical analysis

Data are presented as mean values ± standard error of the mean (SEM). Statistical analysis was performed using Origin Pro 2016 software by one-way ANOVA followed by the Tukey post-test. Differences were considered statistically significant when *p* < 0.05.

## Results

**Fig. 1:**
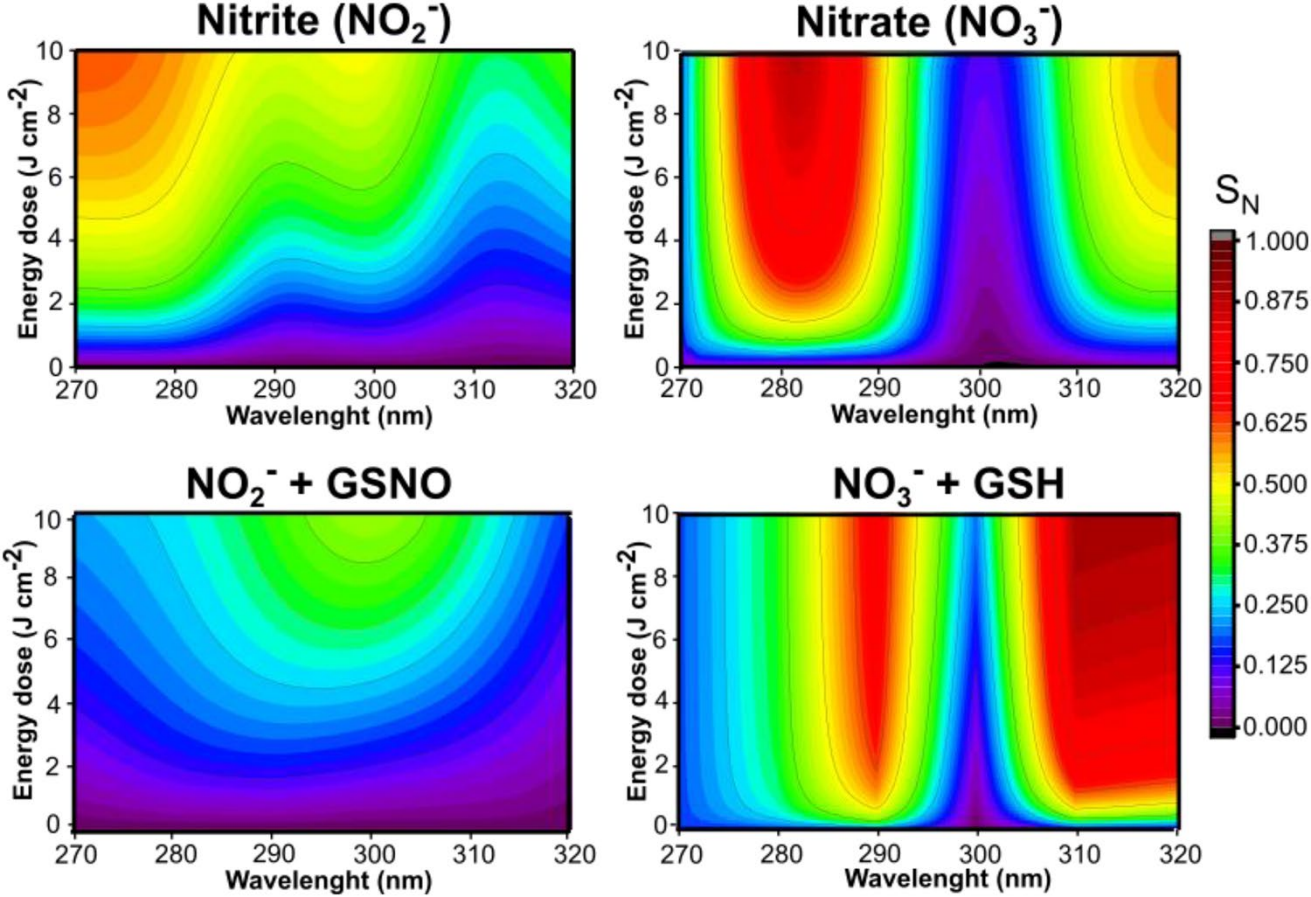
Contour maps of NO generated from irradiated aqueous solutions of (i) NO_2_^−^ (0.05 mmol L^−1^, pH 4.5), (ii) NO_3_^−^ (2.0 mmol L^−1^, pH 4.5), (iii) a mixture of NO_2_^−^ (0.05 mmol L^−1^, pH 4.5) + GSNO (1.0 mmol L^−1^, pH 4.5), and (iv) a mixture of NO_3_^−^ (2.0 mmol L^−1^, pH 4.5) + GSH (1.0 mmol L^−1^, pH 4.5). The measurement of NO generated was performed using the NO meter with an electrochemical sensor. SN is the normalized signal of free NO generated. The colors red, orange and yellow indicate high levels of NO, the green color indicates an intermediary level of NO, and the colors dark blue, light blue and purple indicate a low level of NO in each tested wavelength (250–330 nm) in function of the applied energy (0 to 10 J cm^−2^)

**Fig. 2:**
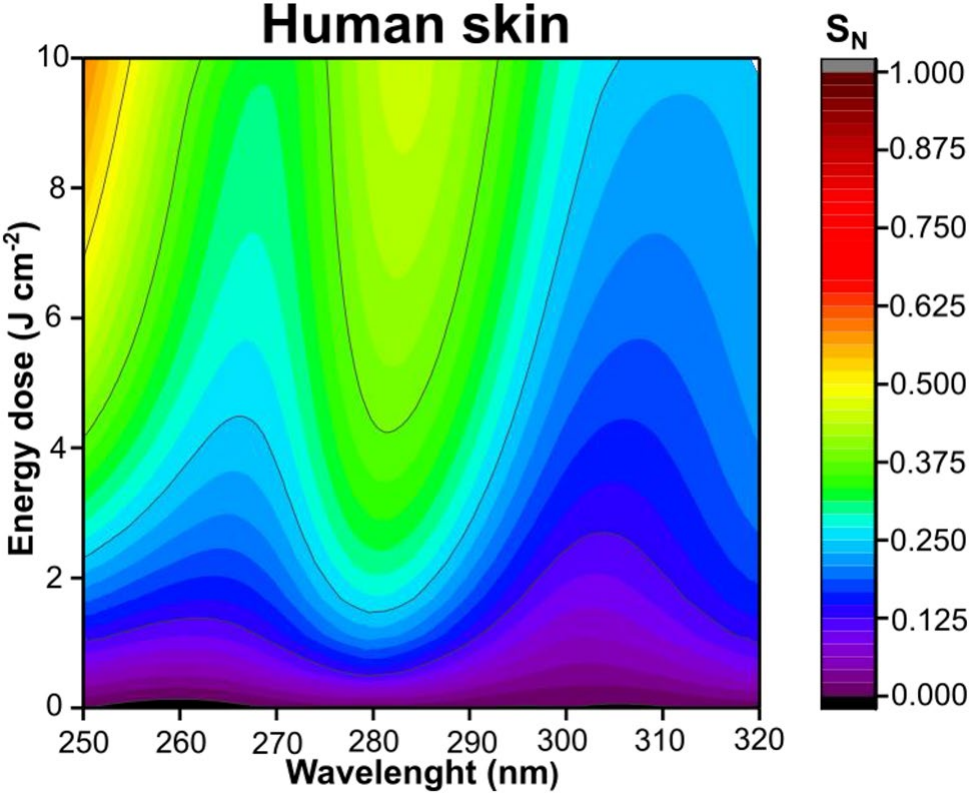
Contour maps of free NO generated from human skin irradiated at 250–320 nm. The measurement of NO was performed using the NO meter with an electrochemical sensor. SN is the normalized signal of free NO generated. The colors red, orange and yellow indicate a high level of NO, the green indicates an intermediary level of NO, and the colors dark blue, light blue and purple indicate a low level of NO in function of the applied energy (0 to 10 J cm^−2^)

In this study, the generation of free NO from aqueous solutions of NO_*x*_ species and human skin was measured in real time upon UV irradiation (270–320 nm), using an NO meter with an electrochemical NO sensor. The formation of NO was measured from irradiated aqueous solutions of: (i) NO_2_^−^ (0.05 mmol·L^−1^, pH 4.5), (ii) NO_3_^−^ (2.0 mmol·L^−1^, pH 4.5), (iii) a mixture of NO_2_^−^ (0.05 mmol·L^−1^, pH 4.5) + GSNO (1.0 mmol·L^−1^, pH 4.5), and (iv) a mixture of NO_3_^−^ (2.0 mmol·L^−1^, pH 4.5) + GSH (1.0 mmol·L^−1^, pH 4.5), which are the most common NO_*x*_ species found in the NO storage in human skin (Fig. 1 and Table 1) The concentration proportions of NO_2_^−^, NO_3_^−^ GSH and GSNO were selected according to in vivo studies (Gamcsik et al. 2012; Zhang et al. 2016). The aim of this study was to mimic in vitro the NO generation from NO_*x*_ species found in human skin. The NO generated from each NO_*x*_ specie (aqueous solutions i–iv) was compared with the levels of NO generated from human skin under the same irradiation condition (human skin slice immersed in water and irradiated) (Fig. 2).

**Table 1:** Description of experimental groups toward composition and concentration, NO release peak and the respective ultraviolet range

Experimental groups	Concentration (mmol L^−1^)	NO release peak (nm)	Ultraviolet range
Nitrite (NO_2_^−^)	0.05	270–275	UVC
Nitrate (NO_3_^−^)	2.0	280–283	UVB
NO_2_^−^ + GSNO	0.05 (NO_2_^−^) + 1.0 (GSNO)	295–300	UVB
NO_3_^−^ + GSH	2.0 (NO_3_^−^) + 1.0 (GSH)	315–320	UVB
Human skin	–	280–285	UVB

In both Figs. 1, 2, it is possible to categorize the intensity of NO generated as high in the hot colors of the contour map (red, orange and yellow) and as low in the cold colours of the contour map (blue and purple). Under UV irradiation, aqueous solutions of NO_2_^−^ and NO_3_^−^ showed a peak of NO formation at 270–275 nm and at 280–283 nm, respectively (Fig. 1). It is possible to notice that NO_2_^−^ and NO_3_^−^ presented different liberations profiles. The highest NO release was for NO_2_^−^ at about 270 nm, however, for NO_3_^−^ the highest liberation was observed at about 280 nm (Fig. 1 and Table 1). The combinations of the species NO_2_^−^  + GSNO and NO_3_^−^  + GSH have high levels of NO generated at 295–300 nm and 315–320 nm, respectively (Fig. 1 and Table 1). It should be noted that the combinations of these species (i.e. NO_2_^−^  + GSNO or NO_3_^−^  + GSH) are not the direct sum of the individual spectra, because multiple reactions between these species might occur. For group NO_2_^−^  + GSNO, the NO_2_^−^ can be reduced to NO upon UV irradiation, however, the presence of GSNO decreased the amount of NO generated in all spectra, therefore this group only reached an intermediate level of NO at ca. 300 nm with a different profile compared to NO_2_^−^ group. (Fig. 1). The interaction of NO_2_^−^ and GSNO need further studies to be better described. It is possible that the GSNO can be decomposed to GSH which is nitrosated by NO_2_^−^, and thus decreasing NO formation. For the group NO_3_^−^  + GSH, the interaction of NO_3_^−^ with thiol groups can have a significant change in NO generation with intermediary reactions leading to other pathways (Cortese-Krott et al. 2014, 2015). Therefore, there are multiple reactions between these species upon UV irradiation.

**Fig. 3:**
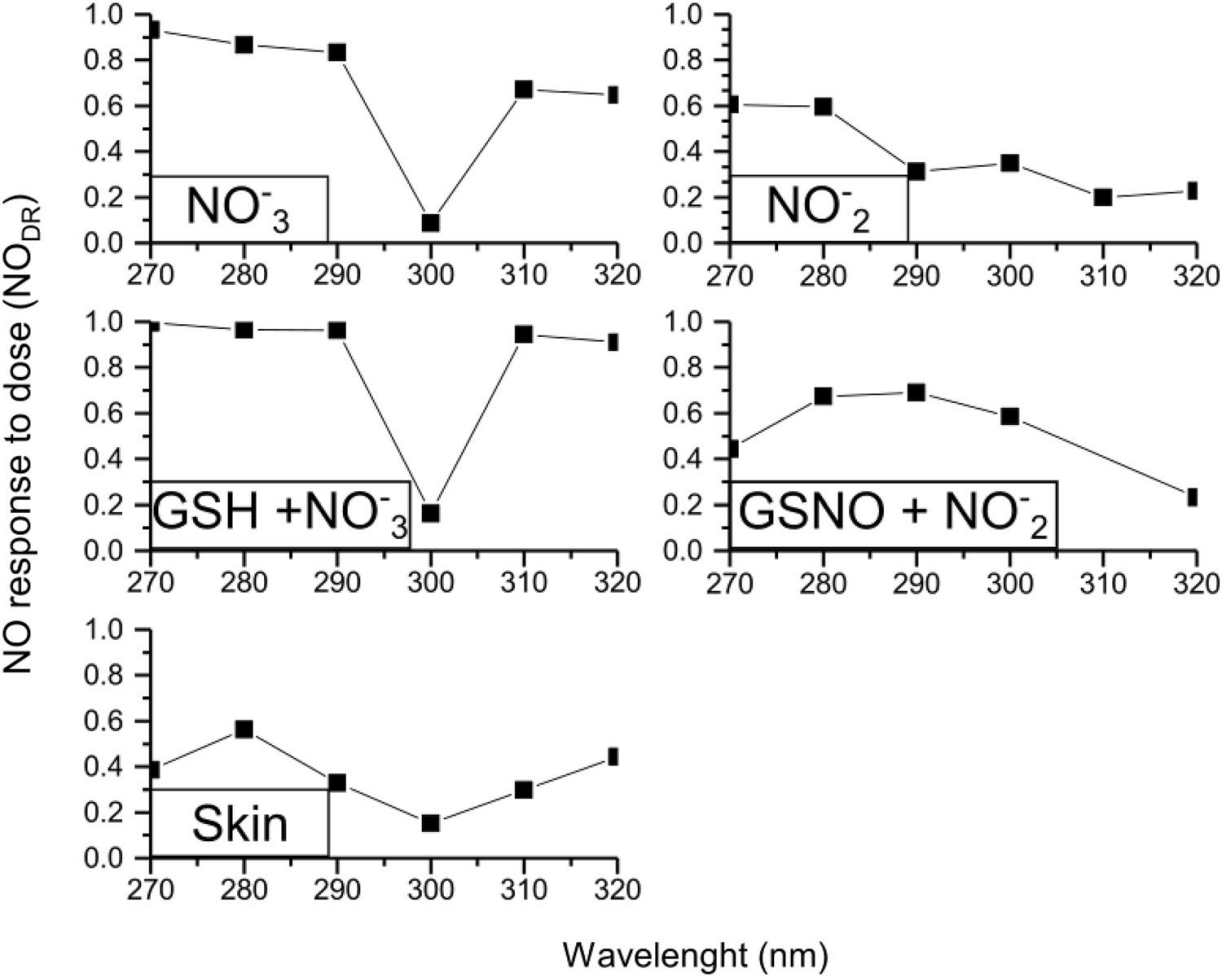
Free NO release response to dose of energy (NODR) from aqueous solutions of (i) NO_2_^−^ (0.05 mmol L^−1^, pH 4.5), (ii) NO_3_^−^ (2.0 mmol L^−1^, pH 4.5), (iii) a mixture of NO_2_^−^ (0.05 mmol L^−1^, pH 4.5) + GSNO (1.0 mmol L^−1^, pH 4.5), (iv) a mixture of NO_3_^−^ (2.0 mmol L^−1^, pH 4.5) + GSH (1.0 mmol L^−1^, pH 4.5), and (v) human skin slice (4 mm). The NODR was calculated through the curve slope of NO signal (SNO) versus energy dose (ED)

We next evaluated the response of NO generated as a function of the applied dose of radiation (NO_DR_) for aqueous solutions of (i) NO_2_^−^ (0.05 mmol·L^−1^, pH 4.5), (ii) NO_3_^−^ (2.0 mmol·L^−1^, pH 4.5), (iii) a mixture of NO_2_^−^ (0.05 mmol·L^−1^, pH 4.5) + GSNO (1.0 mmol·L^−1^, pH 4.5), (iv) a mixture of NO_3_^−^ (2.0 mmol·L^−1^, pH 4.5) + GSH (1.0 mmol·L^−1^, pH 4.5), and (v) human skin slice (4 mm) (Fig. 3). This calculated parameter (NO_DR_) varies between zero and one. For NO_DR_ values closer to zero, the response to the irradiation dose is very slow, in contrast, for NO_DR_ values closer to 1, the maximum NO generation is achieved very fast. This was calculated to each NO_*x*_ specie, at a given wavelength. In Fig. 3 it is possible to notice that the skin slice presented a low response of NO generation to NO_DR_ when compared with aqueous solutions of NO_*x*_ species. This effect might be assigned to the reactions between the chemical species in the skin or even with the interaction of NO with the NO storages (NO_*x*_ species) in the skin, indicating that the skin presented a discrete response. For the human skin slice irradiated in vitro, the highest response to NO generation was observed at 280 nm and the lowest level of NO generation was observed at 300 nm.

**Fig. 4:**
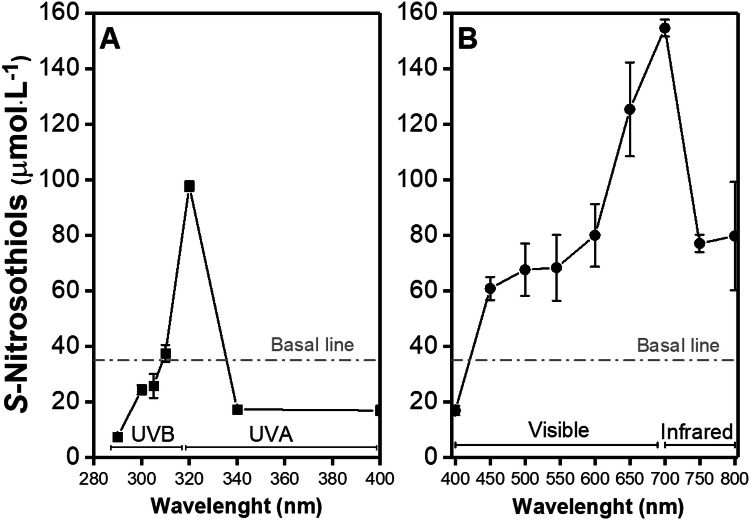
Presence of RSNOs on human skin upon light irradiation divided in **a** ultraviolet irradiation (290–400 nm) and **b** visible (401–700 nm) and infrared (701–800 nm) irradiation. The grey dotted line represents the S-nitrosothiols basal level in the healthy human skin at 35.14 μmol L^−1^

Figure 4 shows the levels of RSNO formation upon in vitro irradiation of human skin slices with 3.0 J cm^−2^ at 280–400 nm (Fig. 4a, UV range) and at 400–800 nm (Fig. 4b—visible and infrared range). The RSNO concentration in unirradiated skin was found to be 35.14 ± 6.32 µmol·L^−1^, which is similar to other reports (Iverson et al. 2018). Figure 4a shows that the irradiation of human skin at 290, 300, 305, 340 and 400 nm decreased the amount of RSNO formation, in comparison with baseline. RSNO is known to be decomposed by the broadband UV and visible light exposure releasing free NO (Sexton et al. 1994). In contrast, the irradiation of skin slices at 310 and 320 nm showed an increase in RSNO levels, in comparison with baseline. Irradiation at 320 nm produced a ca threefold peak of RSNO formation of 97.68 ± 1.80 µmol·L^−1^ (*n* = 8) (Fig. 4a).

Figure 4b showed an increase in RSNO levels for skin irradiated at wavelengths higher than 400 nm with a peak at 700 nm. The values of 450, 500, 550, 600, 750 and 800 nm caused a steady-state RSNO level in skin at ca. 72.25 ± 7.81 µmol·L^−1^, which is twofold higher than the basal line (skin in the dark). The peak at 700 nm had an RSNO level of 154.54 ± 3.06 µmol·L^−1^, which is ca. 4.4-fold higher than the basal line. This increase has an interesting application because it implies that our NO_*x*_ storage in the skin can be replenished by the sunlight, which can increase the NO levels in circulatory system.

Levels of RSNO generated in skin slices rose higher on irradiation with visible light (ca. 700 nm) than UV light (Fig. 4b). The clinical application of the near-infrared light would require a larger amount of time (hours of application per day), which could not fit a clinical trial protocol. In contrast, the UV light irradiation of human skin has a burst of RSNO generation, which could be better fitted for clinical applications.

**Fig. 5:**
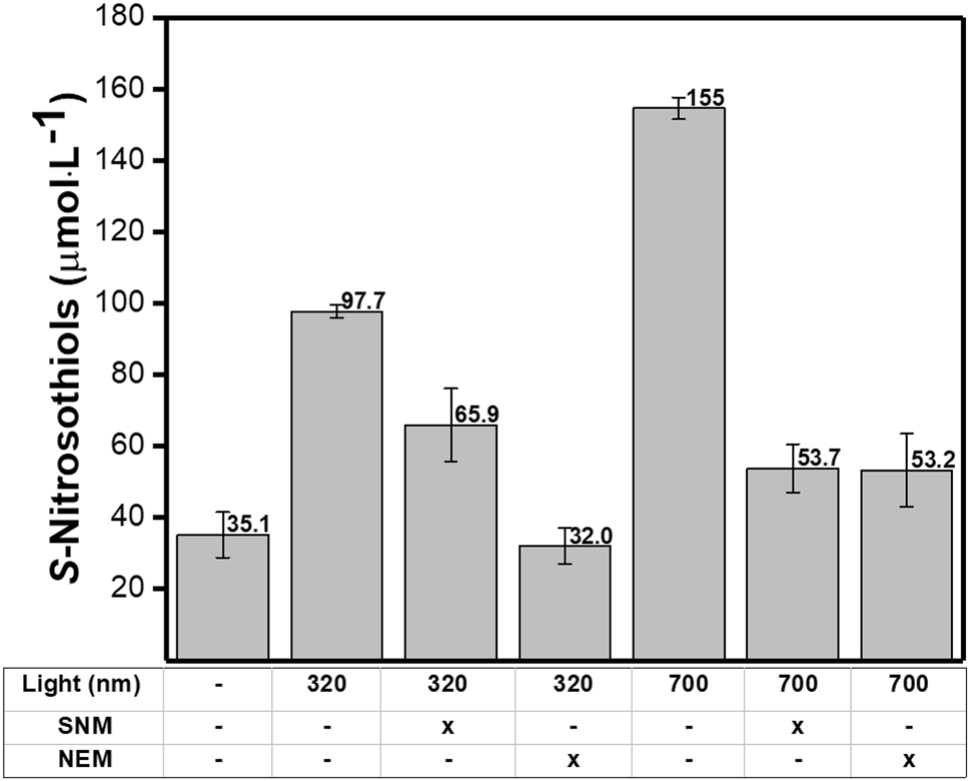
S-nitrosothiols concentrations in human skin after irradiation at 320 nm (UVA) and at 700 nm (Visible) in comparison with the control group (skin in the dark). The skin samples were preincubated with sulfanilamide (SNM) or *N*-ethylmaleimide (NEM) both at 0.5 mmol L^−1^ for 1 h at 25 °C in the dark before the irradiation. SNM is a NO_2_^−^ scavenger and NEM is thiol scavenger

To investigate the formation of RSNOs in irradiated human skin, skin slices were pre-treated with sulfanilamide (SNM) or with *N*-ethylmaleimide (NEM), NO_2_^−^ and thiol (SH) scavengers, respectively. Figure 5 shows the amounts of RSNO formation in human skin slices pre-treated with SNM or NEM, followed by irradiations at 320 or 700 nm. It can be seen that skin pre-treated with SNM decreased the RSNO levels by 30% and 66%, after irradiations at 320 and 700 nm, respectively. Skin slices pre-treated with NEM decreased the RSNO levels by 60% and 66% after irradiations at 320 nm and 700 nm, respectively.

**Fig. 6:**
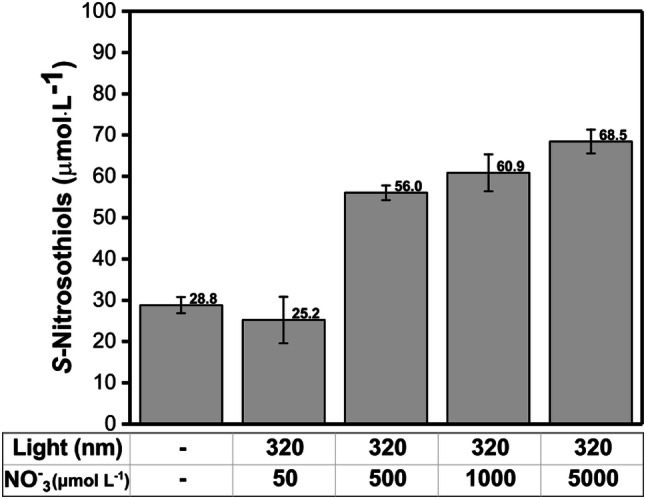
S-nitrosothiols concentration in human skin after irradiation at 320 nm (UVA) in comparison with the control (dark). The skin samples were pre-incubated with a solution of NaNO_3_ at different concentrations (50, 500, 1000 and 5000 μmol L^−1^) for 1 h at 25 °C in the dark

Figure 6 shows the RSNO formation in human skin slices pre-treated with NO_3_^−^ at different concentrations (50, 500, 1000 and 5000 µmol·L^−1^) and irradiated at 320 nm (3 J·cm^−2^). The RSNO levels in the skin increased in a dose-dependent manner with the NO_3_^−^ concentration used in the pre-treatment. This result indicates that the increase of NO_3_^−^ levels in human skin might contribute positively to the RSNO formation pathways.

Thus, these findings show that UV irradiation can increase RSNO formation in human skin using NO_*x*_ stores (NO_2_^−^, NO_3_^−^ and RS^−^) as NO sources. In addition, the observed increase in the concentrations of RSNO in human skin upon UV irradiation might find beneficial effects in the overall cardiovascular system.

## Discussion

NO is an important molecule in the homeostasis of human skin and overall health and involved in important physiological pathways such as vasodilation and macrophage toxicity (Wright and Weller 2015; Weller 2016). NO has a short half-life in the body of 0.05–1 ms (Iverson et al. 2018). Thus in human skin NO is stored in the form of more stable species such as NO_2_^−^, NO_3_^−^ and RSNOs, usually referred to as NO_*x*_ species (Mowbray et al. 2009; Liu et al. 2014). These species are found in significantly higher quantities in human skin compared with other parts of the body, such as the circulatory system, where NO helps regulate blood pressure (Mowbray et al. 2009). Nitrate levels are two to threefold higher in human skin than the circulation (Paunel et al. 2005; Liu et al. 2014). To release NO from NO_*x*_ storage species found in skin, light has an important role.

UV light can trigger the generation of NO from its storage (NO_2_^−^, NO_3_^−^ and RSNOs) in human skin. NO generated from NO_2_^−^, NO_3_^−^ and RSNOs in human skin upon irradiation with UV light can be mobilized from the skin tissue to the bloodstream causing vasodilation and decreasing the blood pressure (Mowbray et al. 2009; Liu et al. 2014; Weller 2016). Indeed, Mowbray et al. 2009 have demonstrated the presence of NOx species (NO_2_^−^, NO_3_^−^, RSNO) in skin surface sweat, human epidermis, and superficial vascular dermis. In addition, the authors showed that human skin exposure to UVA light for 30 min led to the generation of NO (Mowbray et al. 2009). Liu et al. have established a relationship between NO levels, cardiovascular diseases, and sunlight exposure. Their major results demonstrated that human exposure to UVA might significantly decrease blood pressure (Liu et al. 2014). In addition, Weller showed the importance of sunlight for the control of cardiovascular diseases through NO generation in skin, a process that is independent of vitamin D (Weller 2016).

The NO release action spectrum in human skin (Fig. 2) showed a high level of NO generated at 280–285 nm. It is interesting to note the two narrow regions of low NO release at 265 and 310 nm. Those regions coincide with the absorption spectra of urocanic acid and melanin, respectively (Gibbsl et al. 1993; Geng et al. 2008; Zonios et al. 2008; Koch and Schwab, 2018; Madkhali et al. 2019). Gibbsl et al. 1993 showed that urocanic acid has an isomerization reaction at 310–320 nm (Gibbsl et al. 1993) and Geng et al. 2008 showed that melanin has a protective role against UVC-induced DNA damage (Geng et al. 2008).

The closest similarity among the groups studied to human skin in the generation of NO upon UV irradiation was found for the aqueous solutions of NO_2_^−^ (group i) and NO_3_^−^ (group ii) (Table 1). These two species are the major contents of NO_*x*_ species in the skin, which may explain their similarity with human skin model. However, the skin is a complex environment with several proteins and other endogenous compounds that can absorb light and allow numerous reactions.

The interaction of NO with thiols has important implications in several physiological processes (Cook et al. 1996). The transnitrosation reaction (Eq. 1) is defined by the transference of NO from an intact RSNO molecule to another thiol-containing molecule. This reaction plays a pivotal role in several cellular pathways (Williams 1999; de Oliveira 2016). Notable examples of endogenous RSNOs are the S-nitroso-hemoglobin (SNO-Hb), GSNO and S-nitrosocysteine (Cys-NO) (Marley et al. 2001; Keszler et al. 2010).

1 $$R^{\prime}{\text{SH }} + {\text{ RSNO}} \to {\text{RSH }} + \, R^{\prime}{\text{SNO}}$$

There is a strong link between RSNO and cardiovascular functions. Hemoglobin (Hb) can interact with NO through its heme group or a cysteine-containing protein in its membrane, which is called BetaCys93 (Angelo et al. 2006). Studies using engineered mice without BetaCys93 protein confirm their essential importance to mammalian ability to oxygenate tissues and overall myocardial functions (Zhang et al. 2016). Remarkably, BetaCys93 is the only residue in Hb that is conserved across mammals and birds (Zhang et al. 2016).

RSNO also have similar effects to NO itself. Several studies have shown that the delivery of RSNOs causes an increase of dermal blood flow (Khan et al. 2003; Seabra et al. 2004; Vercelino et al. 2013), sustained decrease in mean arterial pressure (Nacharaju et al. 2012), and antimicrobial effects (Kim et al. 2015; Seabra et al. 2016; Pelegrino et al. 2018).

**Scheme 1:**
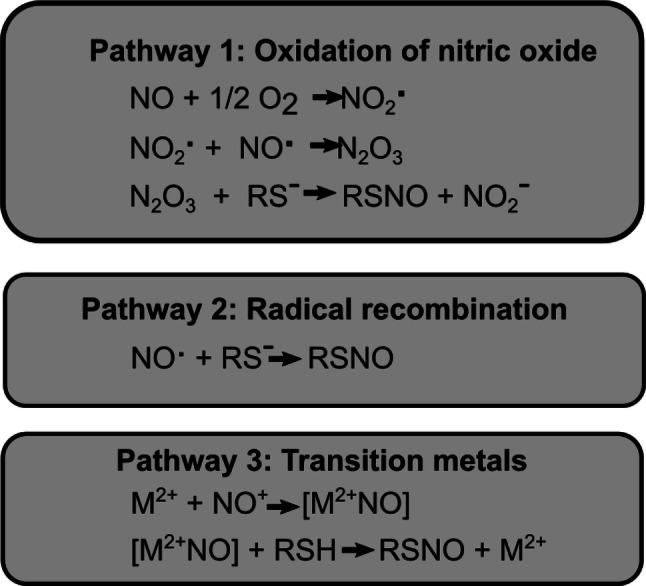
Main pathways of S-nitrosothiols (RSNO) formation in a biological environment

The formation of RSNOs requires NO and is favored by high NO concentrations (Ford and Lorkovic 2002; Martínez-Ruiz et al. 2011). To form RSNO in vivo, there are three main pathways (Scheme 1): (1) NO oxidation pathway, which involves the NO oxidation to peroxynitrite (ONOO^−^) followed by the formation of dinitrogen trioxide (N_2_O_3_), which is a strong nitrosating agent; (2) radical recombination, which involves the NO radical reaction with RS^−^ groups. This mechanism can be favored at low O_2_ environment;(3) transition metals as catalysts, which involves the binding of NO to a transition metal followed by the RSNO formation and metal reduction (Ford and Lorkovic 2002; Smith and Marletta 2012; Wynia-Smith and Smith 2017) (Scheme 1).

There are several reports showing that irradiation of RSNO cleaves the S–N bound, promoting the decomposition of RSNO and the release of NO (Seabra et al. 2004; Taladriz-Blanco et al. 2013). It has been shown that RSNO decomposition may be associated with heterolytic as well as homolytic cleavage of RSNO, which can produce NO^+^, NO^.^ and NO^−^ species (Deliconstantinos et al. 1997; (Williams 1999; Noble and Williams 2000).

The NO half-life depends largely on its microenvironment, the presence of bioreactive molecules, such as O_2_^−^ and HbO_2,_ which can react with NO causing its oxidation (Iverson et al. 2018). In the same way, RSNO decomposition or formation largely depends on its microenvironment. Higher concentrations of NO_*x*_ species (i.e. NO_2_^−^, NO_3_^−^ and RSNO) might generate NO, whereas microenvironment with high concentrations of NO favors the RSNO formation. In addition, the peak observed at 320 nm is related to the increase of one electron oxidation of iron and iron proteins (Zhang and Hogg 2004; Lente et al. 2009).

Zhelyaskov et al. and Sexton et al. showed that depending on the conditions of the experimental test, light can either sharply decrease or stimulate NO concentration (Sexton et al. 1994; Zhelyaskov et al. 1998). Other authors have shown similar results with RSNO formation upon UV irradiation. Deliconstantinos et al. demonstrated that the irradiation of UVB light on human keratinocytes and endothelial cells generated nitrogen oxide species (NO_*x*_), such as NO itself, as well as ONOO^−^ from l-arginine (Deliconstantinos et al. 1997). They also showed an increase of RSNO formation by 4.5- and 3.0-fold after irradiation (290–320 nm with a peak at 312 nm) at 50 mJ·cm^−2^ for keratinocytes and endothelial human cells, respectively (Deliconstantinos et al. 1997). The RSNO formation was inhibited by 60% in keratinocytes and 86% in endothelial human cells by the pre-treatment with monomethyl-*l*-arginine (*l*-NMMA), an *l*-arginine scavenger, measured after irradiation. That indicates the importance of l-arginine in the formation of RSNO, in addition to NO itself (Deliconstantinos et al. 1997). Oplander et al. also described a consistent strong increase in RSNO levels in human skin after UVA light irradiation (340–400 nm with a peak at 366 nm) at 25 J·cm^−2^ detected by Western blot using a S-nitrosocysteine-specific antibody. Their results showed an increase of 2.3-fold in the RSNO levels after the UVA irradiation, in comparison with the basal line (Opländer et al. 2009).

The mechanism of RSNO formation upon UV irradiation may involve a combination of NO oxidation, radical recombination reactions, and transition metal catalysis pathways. The oxidation of NO is a third-order kinetic and it can cause the oxidation of Fe(II) to Fe(II) concomitant with NO oxidation to NO_3_^−^ (Ford and Lorkovic 2002). The UV light can also change the oxidation state of iron, favoring RSNO formation (Pourzand et al. 1999; Karlsson et al. 2006). There is a considerable biological interest in NO reactions with ligands coordinated to a redox-active metal since Hb is an important sink of NO in the cardiovascular system (Pawloski et al. 2001; Angelo et al. 2006; Zhang et al. 2016). Oxidative stress may form a nitrosyl complex that can serve to activate the coordinated NO towards an electrophilic attack and thus cause RSNO formation (Ford and Lorkovic 2002). Therefore, the generation of RSNO in human skin upon UV light and visible light exposure required NO_3_^−^ and SH species. The results indicated that the UV formation of RSNO has a peak at 320 nm and that the RSNO formation requires NO_2_^−^ and thiol-containing molecules. Moreover, visible light formation of RSNO has a peak at 700 nm and the possible mechanism for RSNO formation requires NO_2_^−^ and thiols. These results illustrate how the microenvironment and the oxidation state of the molecules can change the RSNO formation reactions.

## Conclusion

The UV irradiation can trigger the NO generation from human skin with a peak at 280–285 nm. In addition, UV irradiation can also stimulate RSNO formation pathways and contribute to increase the NOx storage in the skin. The main sources for RSNO formation in human skin upon light irradiation are NO_2_^−^, NO_3_^−^ and thiol groups. The RSNO levels may have a relevant role in the vasodilation action of NO. These results can have important implications for clinical trials and light therapy.

## Electronic supplementary material

Below is the link to the electronic supplementary material.

Supplementary file1 (PDF 58 kb)
