# Supplementary material for: Photochemistry of nitric oxide and S-nitrosothiols in human skin
Source: Histochem Cell Biol. 2020 Mar 11;153(6):431–41. doi: 10.1007/s00418-020-01858-w (PMC7300104; doi:10.1007/s00418-020-01858-w)
Supplement: Supplementary file 1 — Supplementary file1 (PDF 58 kb) [file 418_2020_1858_MOESM1_ESM.pdf]

Supplementary information

Methodology

Mathematical analysis of data collected in section 2.5

The free NO release analyses were collected using an electrochemical sensor attached to the NO meter (described in section 2.5). This analysis reported the free NO signal in pico amperes. For groups of (i) NO_2^- (0.05 mmol·L^{-1}, pH 4.5), (ii) NO_3^- (2.0 mmol·L^{-1}, pH 4.5), (iii) a mixture of NO_2^- (0.05 mmol·L^{-1}, pH 4.5) + GSNO (1.0 mmol·L^{-1}, pH 4.5), (iv) a mixture of NO_3^- (2.0 mmol·L^{-1}, pH 4.5) + GSH (1.0 mmol·L^{-1}, pH 4.5), and (v) skin in aqueous media, the NO signal (S_{NO}) was acquired as a function of the applied energy dose (ED) at a given wavelength. This data showed that for a same wavelength the liberation of NO presents an initial linear growth and subsequent saturation at a given signal intensity (S_{max}). To understand better this behaviour, Equation 1 (Michaelis-Menten equation) was adjusted to the collected data. This equation represents the Michaelis-Menten model and S_c stands for the calculated signal and ED_m for the energy dose in which S_c=S_{max}/2.

$$S_c = \frac{S_{max} \cdot ED}{ED_m + ED} \qquad \text{Eq. 1}$$

The adjusted equations presented the determination coefficient (R^2) of no less than 0.96 presenting good fit to the data. As the focus of this study is to analyse the variation of NO production with light irradiation and not to quantify the amount of NO, the S_c was normalized by dividing it by S_{max}, and consequently reported as S_n for each wavelength and calculated using for the energy dose interval from 0 to 10 J·cm^{-2} for every experimental sample group. This analysis

allowed to create a contour maps showing the intensity of NO release as a function of the wavelength of ultraviolet light irradiated for every NO forming chemical species analysed. In addition, to evaluate the dose response of the irradiation in the NO release of the chemical species evaluated it was considered that until ED_m the curve of S_n versus ED presented linear behaviour. By this consideration the angular coefficient is given by $(S_n/2)/ED_m$ and the angle between this line and the ED axis is given by the inverse tangent arc of this coefficient. By these considerations the NO response to dose (NO_{DR}) was calculated dividing the obtained angle by 90º so that if NO_{DR} approaches zero the response to the irradiation dose is null and if it approaches to one it is the maximum as possible for each chemical at a given wavelength.
